# Anterior substitutional urethroplasty using a biomimetic poly‐l‐lactide nanofiber membrane: Preclinical and clinical outcomes

**DOI:** 10.1002/btm2.10308

**Published:** 2022-03-11

**Authors:** Lujie Song, Kunxue Deng, Wei Yuan, Jing Zhang, Jiahao Lin, Xiaoyong Hu, Jianwen Huang, Kaile Zhang, Haitao Zhang, Jiemin Si, Hongbin Li, Tao Xu, Qiang Fu

**Affiliations:** ^1^ Department of Urology Shanghai Jiao Tong University Affiliated Sixth People's Hospital, Shanghai Oriental Institute for Urologic Reconstruction Shanghai China; ^2^ Department of Research and Development Medprin Regenerative Medical Technologies Co., Ltd. Guangzhou China; ^3^ East China Institute of Digital Medical Engineering Shangrao China; ^4^ Biomanufacturing Center, Department of Mechanical Engineering Tsinghua University Beijing China; ^5^ Department of Precision Medicine and Healthcare Tsinghua‐Berkeley Shenzhen Institute Shenzhen China

**Keywords:** biomimetic, poly‐l‐lactide, substitutional urethroplasty, urethral reconstruction, urethral stricture

## Abstract

The aim of this study is to investigate the feasibility and efficacy of a novel biomimetic poly‐l‐lactide (PLLA) nanofiber membrane in repairing anterior urethral strictures from both preclinic and clinic. Biomimetic PLLA membrane was fabricated layer by layer according to the structure of human extracellular matrix. Microstructure, tensile strength, and suture retention strength were fully assessed. Before the clinical application, the safety and toxicology test of the biomimetic PLLA membrane was performed in vitro and in experimental animals. The patients underwent urethroplasty used dorsal onlay or lateral onlay technique. Then, they were followed up for 1 month, 3 months, 6 months, and then annually after the surgery. The mechanical experiments showed well property for application. Biomimetic PLLA membrane was safe according to the in vitro and animal studies. Then, a total of 25 patients (mean age 48.96 years) were included in the study from September 2016 to December 2018. After a mean follow‐up of 33.56 months, 20 patients successfully treated with biomimetic PLLA membrane. Five patients (2 bulbar and 3 penile) suffered postoperational urethral stricture recurrence. None of infection or urinary fistula or any other adverse events related to the use of biomimetic PLLA membrane were observed during the follow‐up period for all patients. The preliminary result confirmed the feasibility and efficacy of the biomimetic PLLA membrane as a novel material for anterior urethral repair. The long‐term effects with more patients should be investigated in further studies.

## 1 INTRODUCTION

Anterior urethral strictures can occur due to various reasons. In China, trauma accounts for the majority of urethral strictures. The incidence of urethral strictures caused by iatrogenic injury has increased.^1^, ^2^ The management of a complex and long‐segment urethral stricture is one of the most challenging issues at present for reconstructive urologists. The most frequently used method for this purpose is substitution urethroplasty using autologous tissues. Various autologous tissues, such as penis skin flaps, bladder mucosa, and oral mucosa, have been proposed for substitution urethroplasty.^3^, ^4^ However, the harvest of these autologous grafts inevitably has led to lesions at the donor site and has limited availability. The use of acellular matrix has provided new ways for urethral repair, such as bladder acellular matrix graft,^5^, ^6^ small intestine submucosa,^7^ and urethral extracellular matrix (ECM).^8^ After implantation, the acellular matrix acts as a scaffold for cell growth and tissue regeneration and provides a suitable microenvironment for cell growth. Finally, it can gradually degrade and be eventually replaced by new tissue. However, the source of human‐ or animal‐derived acellular matrix material is relatively limited. Moreover, the potential risks of ethics, transmission of disease, and immunological problems have aroused concerns.

Poly‐l‐lactide (PLLA) is a kind of synthetic polymer, which approved by the European Union and FDA for medical use. The good biocompatibility, controllable mechanical properties, degradation rate, and topological microstructure make PLLA an extraordinary promising synthetic biomaterial in tissue regeneration. Various manufacture technologies had been applied on PLLA scaffolds preparation. There is a difference between the microstructure of human tissue and traditionally processed PLLA membrane, such as weaving, casting, and hot pressing membranes, which have relatively poorer elasticity, flexibility, cell adhesion and infiltration. The PLLA membrane used in the present study was prepared via additive manufacturing technology. And it is composed of biomimetic PLLA fibers that resemble the ECM in humans. The novel ECM structure has shown good regeneration capacity in tissue repair. The biomimetic PLLA membrane has been preliminarily applied in dura mater repair and complex wound healing.^9^, ^10^, ^11^ However, only few bioactive materials finally applied in clinic, especially in urethral reconstruction field. In the present study, we aimed to investigate the preclinical and clinical outcomes of the novel biomimetic PLLA fiber graft in anterior urethral substitutional urethroplasty.

## 2 MATERIALS AND METHODS

### 2.1 Materials and characterization

The biomimetic PLLA nanofiber membrane used in this study was provided by Medprin Regenerative Medical Technologies Co. Ltd. (Guangzhou, China), which were manufactured through additive manufacturing technology as described in previous report.^10^ Briefly, bioresorbable PLLA fibers were fabricated and deposited layer by layer to form the fiber‐structured membrane, which resembled the extracellular matrix of human soft tissue.

The microstructure of the nanofiber membrane was observed using the scanning electron microscope (SEM, JEOL JSM‐5600LV, Japan). Membrane was cut into small pieces and attached on conductive carbon tape, and gold‐spraying was processed prior to SEM observation. The thickness of the membrane was tested using a commercial hand‐held thickness gauge. Pore size and pore size distribution was tested using automatic mercury porosimeter (MA‐3000, NIPPON, Japan). The tensile strength and suture retention strength were evaluated using a universal material testing machine (Instron 5567, Instron, USA) to represent the mechanical properties of membrane. Tensile strength was examined according to ISO 527‐3 standard. Membranes were soaked in distilled water for at least 1 min to fully hydrate and then cut into stripes with length of 6 cm and wide of 1 cm prior to the mechanical test. Samples with six duplicates were tested with an extension rate of 200 mm/min, and the tensile strength at break was recorded by the machine. Suture retention strength of samples with both dry and wet state was tested following ISO 7198 standard. Membranes were hydrated in distilled water for at least 1 min to form the wet state. NO. 4‐0 sutures (VICRYL, Ethicon, part of the Johnson & Johnson Medical Devices Companies, USA) were used to thread through the samples and tied a knot to form a circle, with 2–3 mm from the sample edge to the semi‐ring. Six samples for each group were tested with a testing speed of 200 mm/min. The stress–strain curves during the testing process were record by the testing machine.

### 2.2 Cell experiments

Human urothelial cells (HUCs, Sciencell, Cat 4320) were used to evaluate the effect of the nanofiber membrane on cell viability, attachment, and morphology. The HUCs were cultured in cell culture dishes in an incubator (37°C, 5% CO_2_) with a humidified atmosphere. Urothelial cell medium (HUM, Sciencell, Cat 4321) was used for cell culture and refreshed every 2 days. Cells at passage 4 were used for the cytotoxicity and attachment experiments.

The sterile membranes were cut into round pieces with diameter of 1 cm and introduced into 48‐well plates. HUCs in 50 μl HUM were added onto the samples with a cell density of 2 × 10^4^ cells per well. Four hours later, 500 μl HUM was replenished into each wells.

Cytotoxicity was evaluated using a Cell Counting Kit‐8 (CCK‐8; Dojindo, Japan) after HUCs seeded on samples and cultivated for 24 h, following the manufacturer's instructions. CCK‐8 working solutions were added into each well to replace the HUM, and incubated at 37°C for 1 h. An enzyme‐linked immune sorbent assay (ELISA) plate reader (Thermo Scientific, Thermo3001, USA) was used to read the absorbance of CCK‐8 reaction solution. Six replicates were prepared for the assay and HUCs seeded directly onto the surface of plate were treated as control group.

Cell attachment morphology was observed using SEM after HUCs cultured on membranes for 4, 8, 24, and 48 h. At each time point, cells on membranes were washed with PBS twice and fixed with 4% paraformaldehyde for at least 4 h. After dehydrated with gradient ethanol and dried, cell morphology was viewed via SEM.

### 2.3 Animal experiments

**FIGURE 1:**
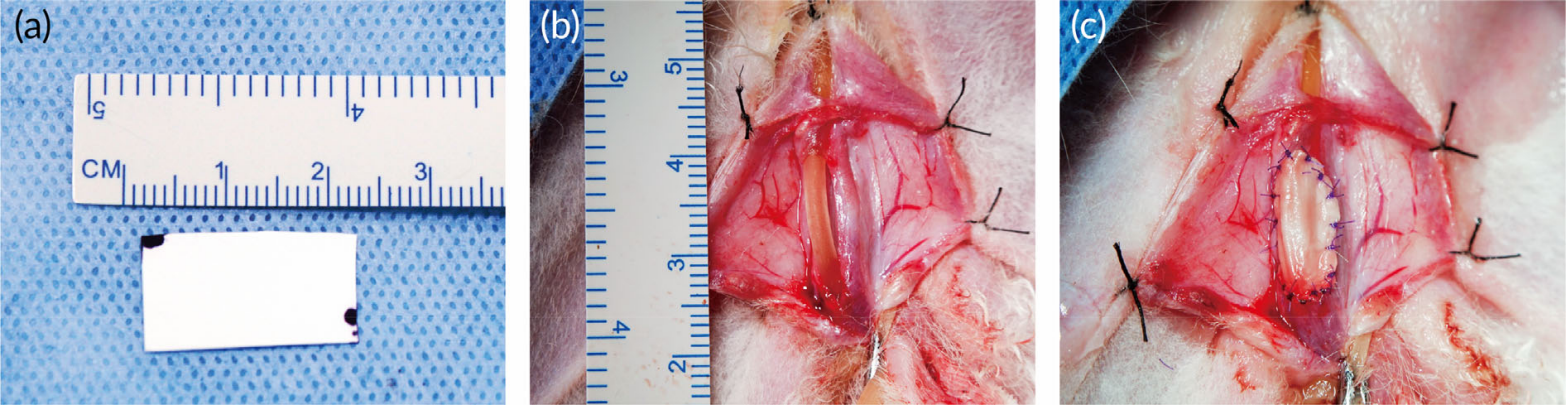
The appearance of biomimetic PLLA membrane (a); 1.5 × 0.8 cm ventral urethral defect was repaired by biomimetic PLLA membrane (b). Then the defect was fixed by biomimetic PLLA membrane (c)

A total of 12 male New Zealand white rabbits (weighing 2.0–3.0 kg) were divided into two groups. Six rabbits were in experiment group and treated with biomimetic PLLA membrane. Other six rabbits were in control group. All surgeries were performed by the same surgeons using procedures described previously.^12^ Briefly, after general anesthesia with intravenous injection of pentobarbital sodium (30–40 mg/kg), the skin approximately 1 cm from the external urethral orifice was opened, the corpus cavernosum and urethra were separated, and a patch (mean length of 1.5 cm and width of 0.8 cm) of the ventral urethra mucosa and the corpus cavernosum were excised. After the establishment of ventral urethra defect model, biomimetic PLLA membrane was sutured to the defect of urethra and corpus cavernosum with 6‐0 absorbable sutures (VICRYL, Ethicon, part of the Johnson & Johnson Medical Devices Companies, USA) (Figure 1). An 8‐French (Fr) silicone catheter was inserted into the urethra, and the wound was closed in layers. For rabbits in control group, just make a ventral urethra defect model. Postoperative evaluations were done for three rabbits from each group at 1 month and 3 months postoperatively, and then, the animals were euthanized. The urethras were incised and harvested for a histology analysis. All the experimental protocols were approved by the Animal Care and Use Committee of Shanghai Jiao Tong University Affiliated Sixth People's Hospital (Approval No. DWSY2015‐0082), and performed in the animal laboratory.

## 3 CLINICAL APPLICATION

### 3.1 Clinical data

**FIGURE 2:**
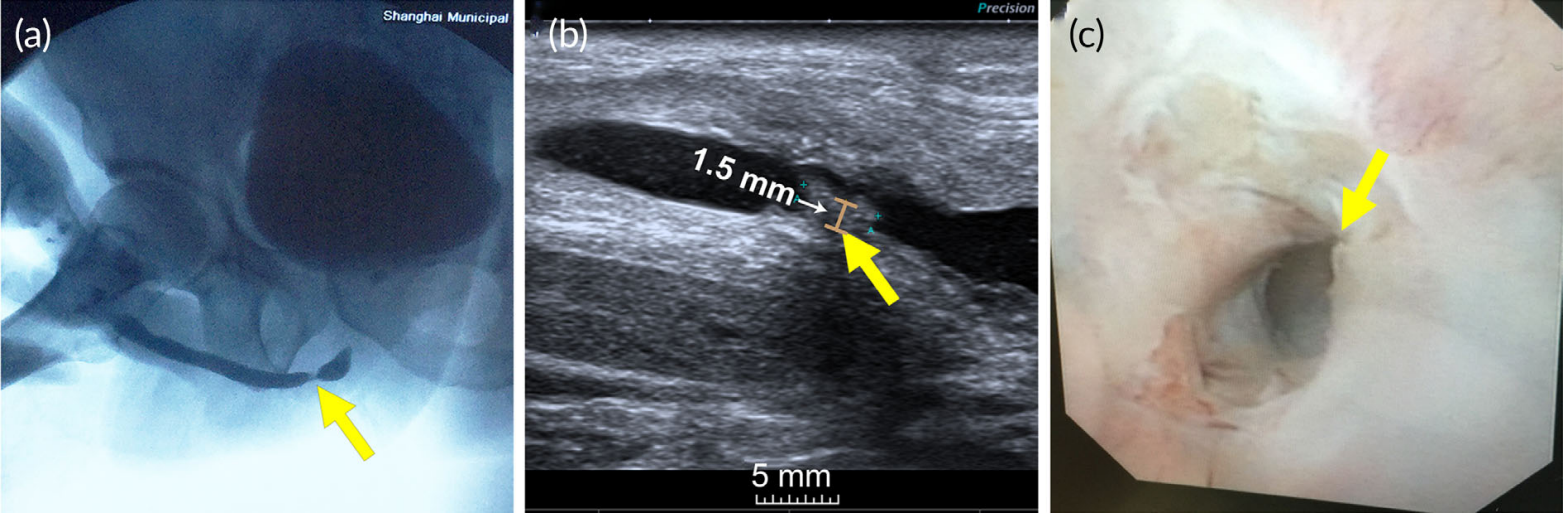
Representative (a) urethrography, (b) urethral ultrasonography, and (c) urethroscopy results of a patient with a bulbar urethral stricture. The yellow arrow indicates the segment of urethral strictures, the brown line indicates the maximum thickness of the urethral scar

A series of male patients who underwent biomimetic PLLA nanofiber membrane urethroplasty due to anterior urethral strictures between September 2016 and December 2018 were reviewed. Several variables related to patient characteristics were recorded, including age, comorbidities, smoking history and etiology, location, prior interventions, and stricture length. Preoperative evaluation included clinical history, physical examination, urine culture, uroflowmetry, urethrography, urethral ultrasonography, and urethroscopy. The inclusion criteria were as follows: the length of anterior urethral stricture was at least 1 cm and less than or equal to 7 cm, anastomotic urethroplasty was not possible, urethral lumen was not completely occluded (evaluated by urethrography or ultrasonography or urethroscopy) (Figure 2). The patients with urine infection were required to accept antibiotic therapy according to the drug sensitivity test until twice consecutive negative urine culture. The clinical use of the biomimetic PLLA membrane for urethral reconstruction in this study was approved by the ethics committee of Shanghai Jiao Tong University Affiliated Sixth People's Hospital (ethics number: 2016‐35).

### 3.2 Surgical procedures

Urethroplasty was performed in all patients using biomimetic PLLA membrane as grafts for the urethral augmentation. Two different procedures were used: dorsal onlay graft augmentation and lateral onlay graft augmentation.

After administering general anesthesia to the patients, they were placed in a lithotomy position. A lengthwise incision from the ventral penis was made for patients with penile strictures, while a midline perineal incision was made for those with bulbar strictures. Then, the urethra was split carefully from the dorsolateral side to expose the stricture segment. The stricture length and width of the residual urethral plate were further measured intraoperatively. An incision of the stricture urethra was made beyond the stricture segment proximally and distally into the normal urethral lumen until the mucous was soft, flexible, ruddy, and shiny, and the 24‐Fr urethral bougie could easily pass through both proximally and distally.

**FIGURE 3:**
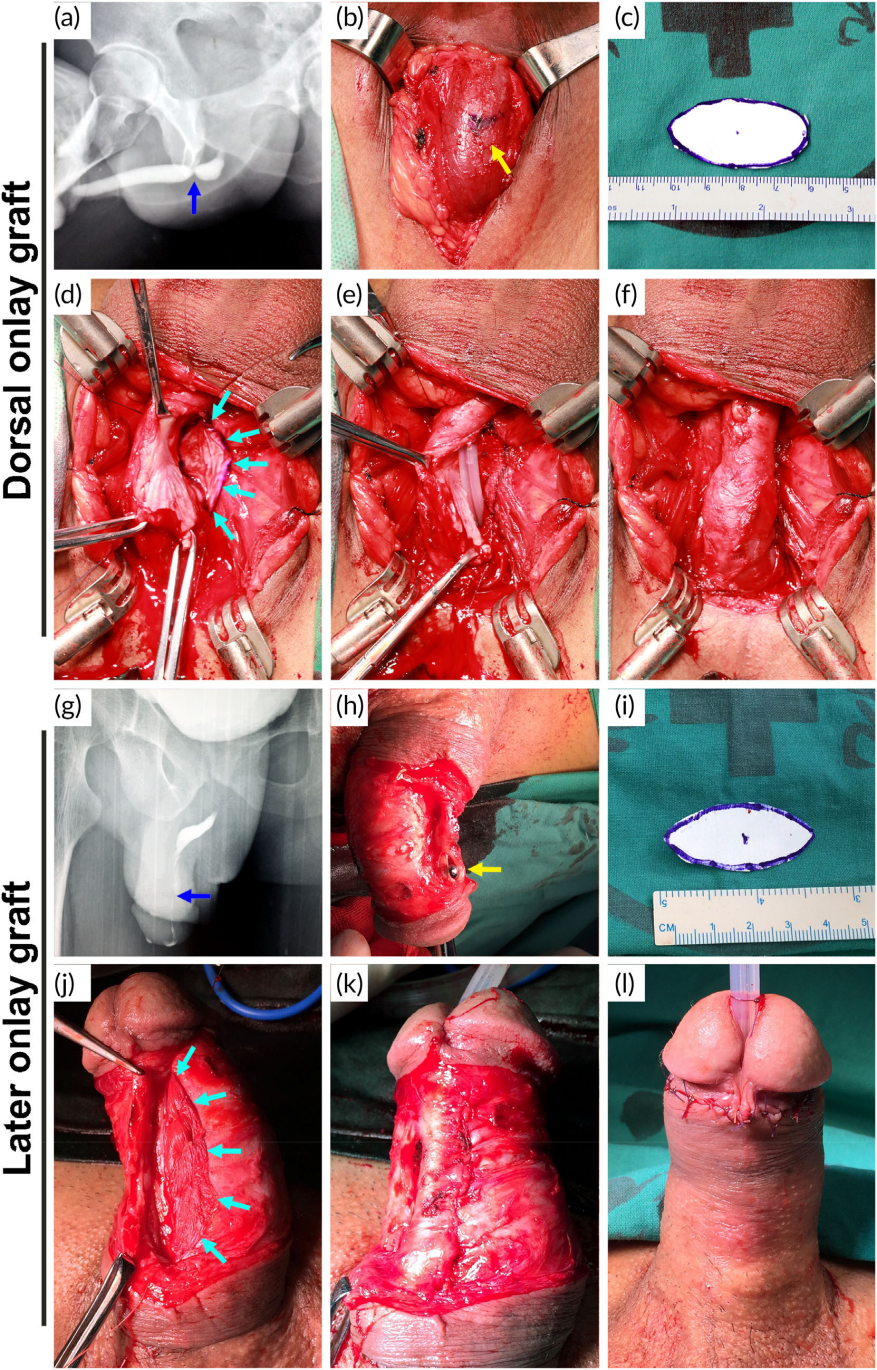
Two different procedures using biomimetic PLLA membrane as grafts for treating a bulbar (a–f) and penile (g–l) urethral stricture. (a and g) Urethrography showing a urethral stricture (blue arrow). (b and h) Dissection of the bulbar or penile urethra (yellow arrow). (c and i) Tailored PLLA membrane. (d and j) PLLA membrane was fixed on the dorsal or lateral surface of the urethral defect (cyan arrow: the edge of the membrane). (e) Silicone catheter was indwelt. (f and k) Restoration of the urethra. (l) Postoperative appearance of the penis

The biomimetic PLLA membrane was cut as needed and immersed in normal saline for 1 min before use. Then, the tailored oval membrane was spread fixed to the corpora cavernosa on the dorsal or lateral side of the urethral stricture segment using 5–0 absorbable sutures (VICRYL, Ethicon, part of the Johnson & Johnson Medical Devices Companies, USA). Subsequently, the membrane or the graft was sutured to the edge of the stricturotomy using continuous sutures. After suturing one side, a 16‐Fr silicone catheter was indwelt in patients with bulbar urethral strictures and a 14‐Fr silicone catheter in patients with penile urethral strictures. Then, the other side was anastomosed. The surrounding dartos fasciae were detached appropriately and made to overlap on each other, and the reconstructed segment of the urethral stricture was covered (Figure 3).

### 3.3 Follow‐up

The patients were followed up after 1 month, 3 months, 6 months, and then annually. Clinical evaluations included uroflowmetry or voiding function report. Urethrography or urethroscopy was performed if the maximum urine flow rate was continuously lower than 15 ml/s or selectively performed before the removal of the suprapubic catheter in some patients. Success was defined as a maximum flow rate of >15 ml/s and without the need for further surgical interventions, such as dilatation or optical urethrotomy. Stricture recurrence was defined as recurrent symptomatic stricture requiring further operative intervention following initial intervention.

## 4 RESULTS

### 4.1 Characterization of physical and mechanical properties

**FIGURE 4:**
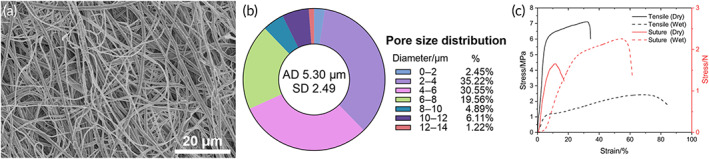
SEM microstructure (a) and pore diameter distribution (b) of the biomimetic PLLA nanofiber membrane. Stress–strain curves of the scaffolds and the suture strength test in dry and wet states (c)

The biomimetic PLLA membrane was composed of a number of nanofibers and had a highly porous network with interconnected pores (Figure 4a). The thickness and average, largest, and smallest pore sizes of the scaffolds were about 315, 5.3, 13.65, and 1.26 μm, respectively (Figure 4b). The breaking strength and elongation at the break of the scaffolds were 7.12 MPa and 32.5%, respectively, in the dry state. In the wet state, the breaking strength and elongation of the composite scaffolds were 2.42 MPa and 70.3%, respectively (Figure 4c). The suture retentions of the scaffolds were 11.8% and 1.66 N in the dry state and 54.5% and 2.26 N in the wet state (Figure 4c). The suture retention strength of the biomimetic PLLA membrane was more than or close to 2.0 N in the wet state, which was generally accepted where suturing was required with tissue in implantation cases.^13^

### 4.2 Cell experiments

**FIGURE 5:**
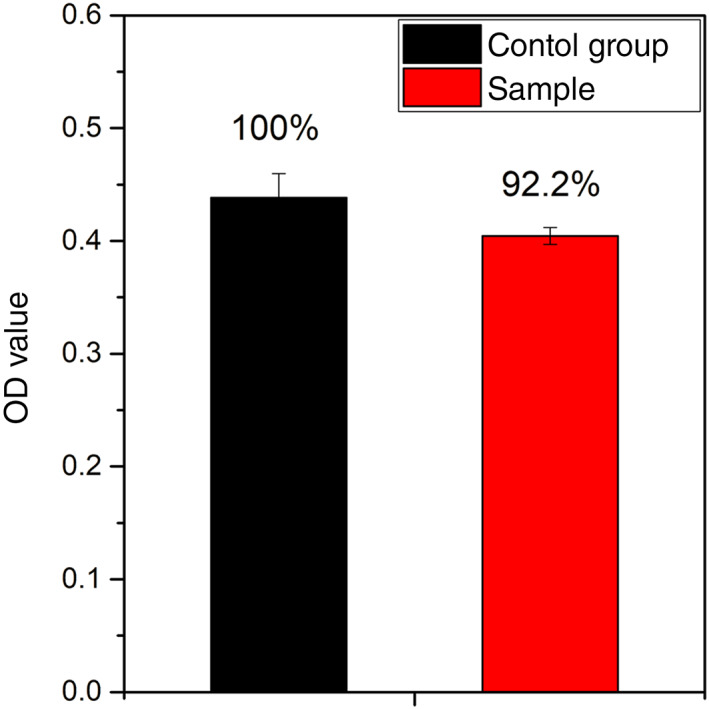
Cytotoxicity of PLLA fiber membrane sample and the control group analyzed using CCK‐8 kit. Cytotoxicity was evaluated using a CCK‐8 after HUCs seeded on samples and cultivated for 24 h

OD values of cytotoxicity experiment analyzed using CCK‐8 kit were shown in Figure 5. There is no significant difference between the nanofiber membrane sample and the control group. And the relatively OD value of sample to the control group was 92.2%, which meant the cytotoxicity level of nanofiber membrane to human urothelial cells was zero.

**FIGURE 6:**
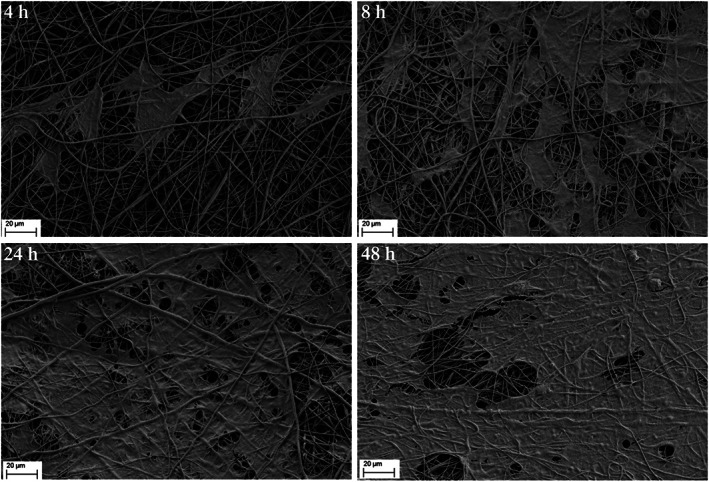
The attachment of HUCs after seeded on PLLA/gelatin nanofiber membranes for 4, 8, 24, and 48 h

The adhesion and distribution of HUCs after seeded on nanofiber membranes for 4, 8, 24, and 48 h were shown in Figure 6. Cell conjugation formed after 48 h of culture. HUCs had a relatively high crawling and proliferation rate on samples.

### 4.3 Animal experiments

**FIGURE 7:**
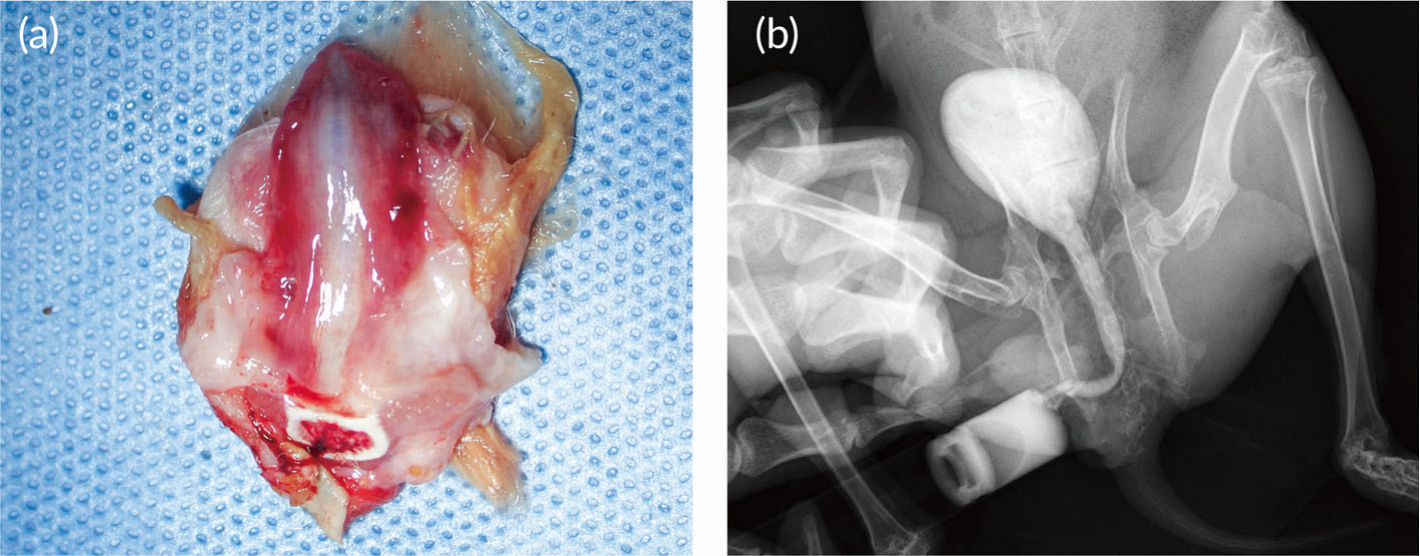
The represent gross observe image of the urethra after 3 months postoperatively in biomimetic PLLA membrane group (a). The reconstructed urethra was dissected longitudinally from the ventral side, and the ruddy areas on both sides represented the reconstructed segment. Urethrography was performed before animals were executed (b)

**FIGURE 8:**
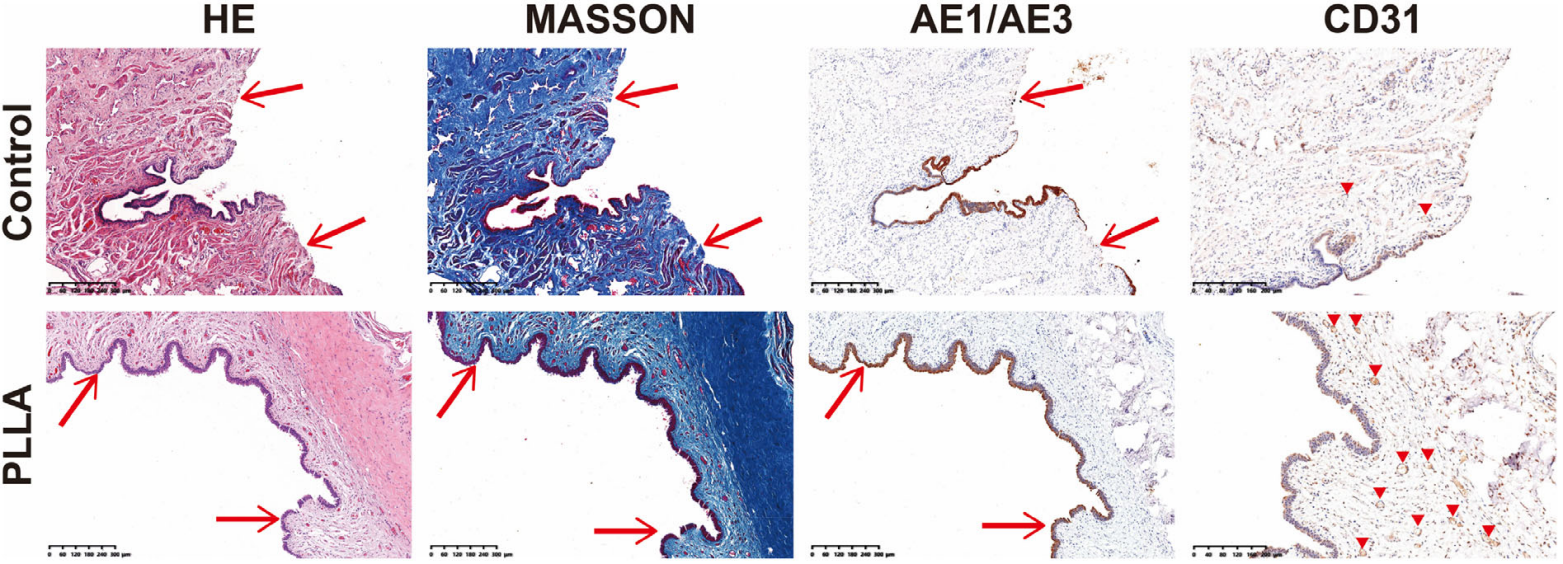
Representative hematoxylin and eosin staining (HE), Masson trichrome staining, AE1/AE3 (expression in the urothelium) immunohistochemical staining, and CD31 (expression in the vascular endothelium) immunohistochemical staining at 3 months after implantation in the two groups. The red arrows indicate surgical sites. The red triangles indicate blood vessels

Twelve rabbits in two groups underwent operations survived until the scheduled euthanasia, and there was no infection at the operative site. Silicone catheter slipped out about 5–7 days after operation. Either 1 month or 3 months after the surgery, all the rabbits in control group suffered variety degrees of urethral stricture. Meanwhile, the urethral stricture was found in one rabbit in the repaired group at 1‐month post‐operation. At 3 months, the retrograde urethrography confirmed the wide lumen of rabbit urethral in the repaired group; an 8‐Fr silicone catheter was successfully inserted into the urethra; and the mucosa looks smooth; the urethra demonstrated normal‐appearing tissue without sign of inflammation, fistula, urethral diverticulum, or stricture (Figure 7). Macroscopic examination demonstrated that there was no evidence of biomimetic PLLA membrane residue. While, in control group, an 8‐Fr silicone catheter was failed to inserted into the urethra. Rabbits in the repaired group showed epithelium coverage at 1 month after operation, but the mucosa was incomplete. At the time of 3 months, histologic assessment using H&E and immunolabeling using AE1/AE3 (expression in the urothelium) confirmed that the continuity of mucosa at the reconstructed site was recovered with the epithelial cells arranging stratified in an orderly manner and appeared like the normal urethra mucosa. At the same time, Masson's trichrome staining and immunolabeling of CD31 (expression in the vascular endothelium) showed the formation of smooth muscle and microvascular in the corpus cavernosum area was apparent. In the control group, there was only few epithelium covered the injured site, and the collagen fibers in the corpus cavernosum were markedly disordered (Figure 8).

### 4.4 Clinical application

Twenty‐five patients were included in the study. The mean age of patients was 48.96 years (range from 19 to 83). Table 1 summarizes the basic clinical characteristics of patients. Out of 25 patients, 16 had bulbar urethral stricture, eight had penile urethral stricture, and one had both bulbar and penile urethral stricture. Lateral onlay technique was used in 13 (52.00%) cases (five bulbar, seven penile, and one both), dorsal onlay in 12 (48.00%) cases (11 bulbar and one penile). The patient suffered both bulbar and penile urethral stricture used two biomimetic PLLA membrane (penile, 3 × 2 cm^2^; bulbar, 3.5 × 2 cm^2^) in surgery.

**TABLE 1:** Patient's characteristics and surgical outcomes

	No. (%)/mean (SD)	
Age (year)	48.96	17.85
Location		
Bulbar	16	64.00%
Penile	8	32.00%
Both	1	4.00%
Etiology of trauma		
Iatrogenic injury	8	32.00%
Trauma	13	52.00%
Idiopathic	4	16.00%
Details of iatrogenic injury		
Intravesical chemotherapy	1	12.50%
Transurethral resection of the prostate	3	37.50%
Urethral calculi	2	25.00%
Urethral catheterization	2	25.00%
Cystostomy		
Yes	10	40.00%
No	15	60.00%
Stricture length (cm)	3.45	1.21
Surgical procedures		
Dorsal	12	48.00%
Lateral	13	52.00%
Size of biomimetic PLLA membrane		
Length (cm)	3.55	1.05
Width (cm)	1.62	0.34
Area (cm^2^)	5.86	2.21
Preoperational maximum urine flow rate (ml/s)	4.16	4.87
Postoperational maximum urine flow rate (ml/s)		
1 month	24.35	6.12
The last time point of follow‐up	33.56	16.50
Outcome		
Success	20	80.00%
Bulbar	14	
Penile	5	
Both	1	
Failure	5	20.00%
Bulbar	2	
Penile	3	
Both	0	
Follow‐up (month)	33.56	16.50

**FIGURE 9:**
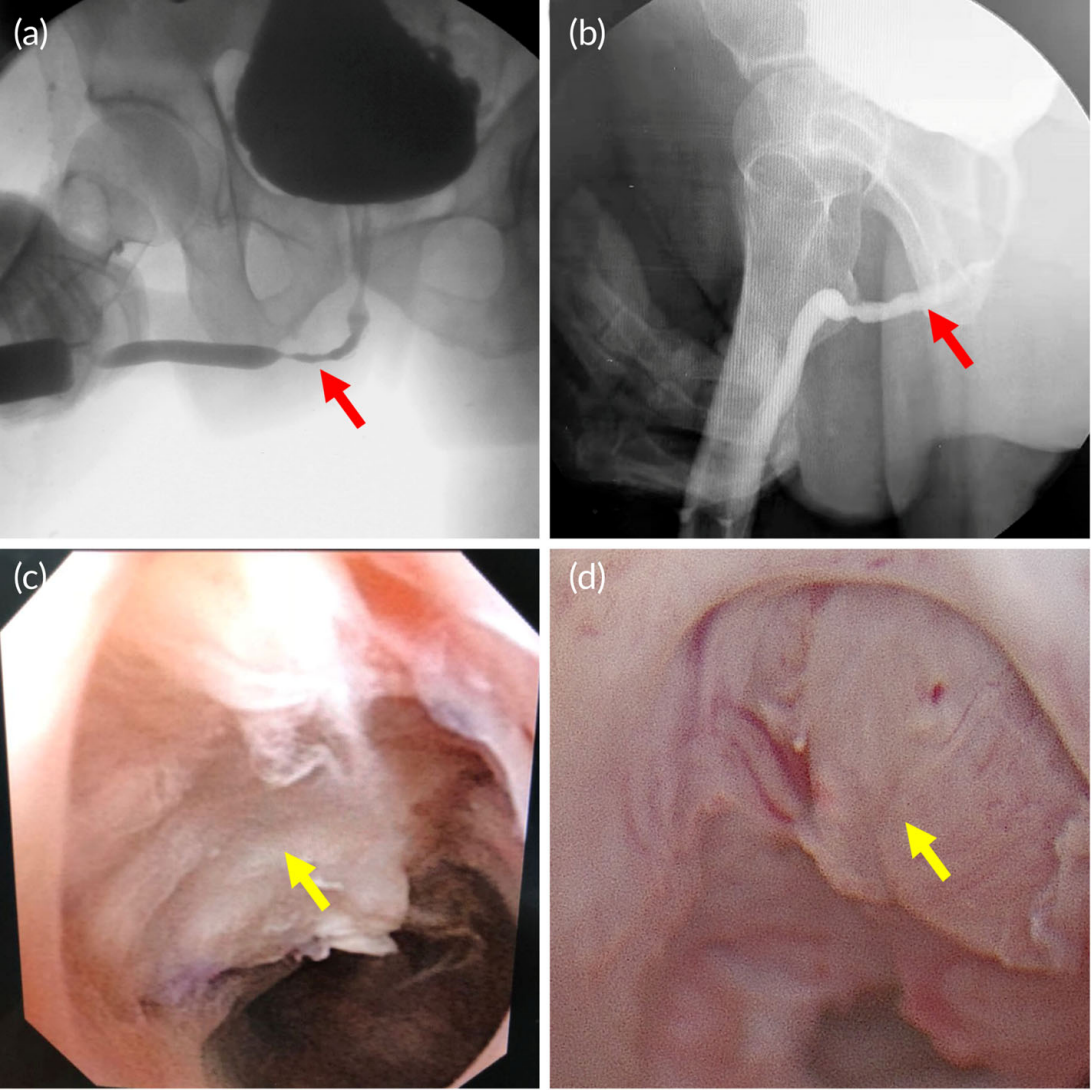
Representative (a) preoperative and (b) postoperative urethrography from patient of biomimetic PLLA membrane group. There were no postoperative leakages of anastomosis, and the urethral lumen was wide and developed normally. Flexible cystoscopy after biomimetic PLLA membrane urethroplasty (c) 2 months and (d) 3 months postoperatively. The proliferation of some villus‐like tissue was found at the substitutional urethral segment. Moreover, the mucosal epithelium looked ruddy, and the lumen of the replacement segment was quite large. The red arrow indicates the site of urethral stricture preoperationly and postoperationly, and the yellow arrow indicates the reconstructed urethra

The catheter was removed 3–4 weeks after the surgery in all patients. The mean follow‐up duration was 33.56 (16.50) months. Compared with the preoperative maximum urine flow rate, the value at 1 month postoperatively was significantly higher (4.16 vs. 24.35, *p* <0.05, statistic method: Wilcoxon Signed Ranks Test). The results showed a high success rate (80.00%). According to the stricture location, the overall success rate was 62.50% in penile urethroplasty, 87.50% in bulbar, 100% in one with both bulbar and penile urethral stricture. The lateral onlay technique showed 80.00% success rate when used in bulbar urethra (four cases) and 57.14% success rate when used in penile urethra (four cases). The dorsal onlay showed 90.91% success rate when used in bulbar urethra (10 cases) and 100.00% success rate when used in penile urethra (one case). The postoperative urethrography (Figure 9a,b) and urethroscopy (Figure 9c,d) had confirmed that the lumen of the replacement segment urethra was wide enough in success ones. Five patients (two bulbar and three penile) suffered postoperational urethral stricture recurrence, the details of patients suffering postoperational urethral stricture recurrence were summarized in Table 2. One of the patients (patient no. 5) refused further intervention and chose urethrotomy after several urethral dilatations but was still followed up closely. Two patients (patient nos. 1 and 4) had normal urination without further intervention after undergoing lingual mucosa graft (LMG) augmentation urethroplasty, the other two patients (patient nos. 2 and 3) had unobstructed voiding after performing urethroplasty using Orandi flap. Besides, none of infection or urinary fistula or any other adverse events related to the use of biomimetic PLLA membrane were observed during the follow‐up period for all patients.

**TABLE 2:** Details of patients suffering postoperational urethral stricture recurrence

Patient no.	Age (year)	Location	Etiology	Cystostomy	Stricture length (cm)	Surgical procedures	Size of biomimetic PLLA membrane (cm)	Time point of failure (postoperation)	Followed treatment
1	36	Bulbar	Urethral calculi	No	4.2	Dorsal	4.2 × 1.8	36 months	LMG augmentation urethroplasty
2	54	Bulbar	Idiopathic	Yes	4	Lateral	4 × 1.5	2 months	urethroplasty using Orandi flap
3	43	Penile	Intravesical chemotherapy	No	5	Lateral	5 × 2	7 weeks	urethroplasty using Orandi flap
4	61	Penile	Trauma	No	3	Lateral	3 × 1	6 weeks	LMG augmentation urethroplasty
5	36	Penile	Idiopathic	No	3	Lateral	3 × 1.5	3 months	Urethrotomy

Abbreviation: LMG, lingual mucosa graft.

## 5 DISCUSSION

Urethral defects or strictures caused by congenital deformity, trauma, or inflammation are common concerns in urology, and substitution urethroplasty is one of the most important treatments. Therefore, the best selection of tissues or materials used in substitution urethroplasty is worth investigating. As the representation of autologous tissue, oral mucosa has been proposed for substitution urethroplasty. The use of oral mucosa may result in several complications. Patients usually suffer from speech impairment and problems with eating and drinking in the early postoperation period; sensitivity disorders may also appear later.^14^, ^15^ Other autologous tissues, including penile flaps and bladder mucosa, may suffer hair growth, tissue contracture, lithogenesis, and diverticulum formation.

There are several studies reported clinical application of tissue‐engineered material in substitution urethroplasty. In 2011, a tissue‐engineered autologous urethra was reported used in patients with urethral defects.^16^ The muscle and epithelial cells were harvest from the patients, expanded, and seeded onto tubularized PGA scaffolds. After a mean 71 months follow‐up, none of the patients suffer the recurrence. This is a great approach in urethral reconstruction. In 2018, a tissue‐engineered oral mucosa graft named MukoCell® was applied in anterior urethroplasty.^17^ It also had an overall high success rate. Unlike these studies, we fabricate the biomimetic PLLA nanofiber membrane via additive manufacturing technology, mimicking the ECM structure of humans. Without harvesting harm, long‐time tissue culture, and bio‐safety problems, synthetic material provides a valid alternative to tissue‐engineered ones owing to their lower costs (up to 10 times less), wider availability, and fewer ethical concerns. Besides, the pore size, construction, and absorbability could be adjusted according to demands. It is a good graft choice for the urethral substitution. Meanwhile, several studies also showed that the ultrastructure and 3D architecture of collagen fibers of the acellular matrix were important in modulating the cells' ability to migrate into the scaffold or influence tissue‐specific cell phenotype.^18^, ^19^ Thus, the exploration and development of a new urethral substitution material that can mimic the structures of an acellular matrix are urgently needed in clinic.

Using additive nanomanufacturing technology to prepare biomimetic scaffolds is realistic with the rapid development of technology in recent years. A biomimetic PLLA nanofiber membrane is sequentially fabricated in layers to form a 3D structure. The cell experiment showed a high biocompatibility (Figures 5 and 6), which is coincident with previous study.^9^, ^10^ Compared with the acellular matrix, the biomimetic 3D structure with a highly adjustable porous network can facilitate the passage and exchange of nutrients and gases, which are important for cellular growth and tissue regeneration.^20^ Besides, the biomimetic PLLA membrane is more flexible and can closely adhere to the tunica albuginea of corpora cavernosum on the dorsal surface of the urethra, with no distortion or wrinkle even after fixed sutures (Figure 3d,g). This result was also confirmed in animal experiment, compared with the bovine skin acellular matrix and silk fibroin, PLLA nanofiber membrane could attached smoothly on the surface of the rabbit bladder tissue (Figure S1). Histological analysis also confirmed the biomimetic PLLA membrane attached smoothly and tightly on the surface of the defect area postoperative 3 days (Figure S3). Moreover, the biomimetic PLLA membrane possesses good mechanical properties and has a more suitable biodegradation rate compared with the acellular matrix. It has also shown good hydrophilicity and can be quite flexible on dipping in the normal saline for 1 min with excellent mechanical strength and enough suture retention strength in the wet state. The use of the biomimetic membrane can help greatly shorten the operative duration and reduce the related complications at the autologous graft donor site.

Before starting the clinical application of biomimetic PLLA membrane, the fully assessment was performed in animal study. The results showed that the reconstructed urethral lumen was wide and smooth, and the epithelial cells arranged orderly. It is safe and ready to use in our patients. Preliminarily, the biomimetic membrane was selected mainly for patients with an anterior urethral stricture without urethral occlusion and severe scars because the healthy spongiosum environment can promote normal urethral regeneration.^21^ One of the patients in this study underwent urethroscopy 3 months postoperatively, we had not observed any PLLA membrane from the surface of urethral mucous (Figure 9d).

In the present study, the patient's characteristics and surgical outcomes were collected, and the result revealed that the biomimetic PLLA membrane had a high success rate in the bulbar urethra (87.50%), lower success rate in penile urethra (62.50%). Besides, the biomimetic PLLA membrane also showed a possibility, which it could be used for patient who suffered both bulbar and penile urethral stricture at same time. However, when the characteristics of patients with failed urethroplasties were analyzed, the failure of the biomimetic PLLA membrane was found mainly to occur in patients with penile urethral stricture.

Sufficient blood supply is beneficial to the surrounding cell infiltration and material degradation. In animal experiment, the biomimetic PLLA membrane was used in bladder substitution. Bladder had a better blood supply, lots of red blood cells, and a few of white blood cells filled in the space of the biomimetic PLLA membrane (Figure S3). This phenomenon might also account for the quick degradation of biomimetic PLLA membrane in animal model. While, in human, the cavernous in bulbar urethra is stronger and thicker than penile urethra, and supplies with more blood. The residual urethral plate appearance of bulbar urethra is normally appearing much better than that of penile urethra after opening the narrowed urethral lumen. The anatomical difference led to the insufficient blood supply of the penile urethra, which might be one reason for the more failed urethroplasties in penile urethral strictures. Besides, the spongiofibrotic degree could be another influence factor for the blood supply. As our center is one of the biggest tertiary referral urethral reconstruction centers in China, before the patients came to our center, some of them had already received several interventions. The prior interventions could contribute a lot to the spongiofibrotic degree. Tissue regeneration was based mainly on the nutrition supply and inosculation of the urethral plate.^22^ Thus, the spongiofibrotic segment of the urethra was not good enough to support the regeneration of urethral epithelial cells inside the lumen, which might be an important reason for the failed urethroplasties in the biomimetic PLLA membrane.

As for polymer materials, PLLA was confirmed to be degraded in vivo. Due to the ethical concern and for human rights protection, biopsy and extra examinations were not performed to analyze the degradation state. However, in the animal experiment, we found the biomimetic PLLA membrane was vanished in the rabbit bladder in 2 weeks, the fixed area showed no significant different in gross observation (Figure S2). Besides, the stress–strain curves of postoperative bladder were also similar to the normal bladder (Figure S5). At the time point of postoperative 3 days, we found the biomimetic PLLA membrane attached smoothly and tightly on the surface of the defect area (Figure S3C,D). The surface of the biomimetic PLLA membrane had been changed significantly compared with Figure 4a (Figure S4). The animal experiment found that the biomimetic PLLA membrane vanished within 2 weeks, it was totally different with the results of in vitro degradation experiment (Figure S6). It also does not mean the biomimetic PLLA membrane could be degrade in 2 weeks in human bladder or urethra, because the internal environment is different in human and animal. The in vivo degradation experiment is more important for bioactive material, especially the one prepared for human application. The degradation state of the biomimetic PLLA membrane in human still needs further evaluation.

In this study, the biomimetic PLLA membrane was used for substitution urethroplasty, which also seemed to be promising in urethra reconstruction. However, this study had some limitations. The sample size was not large enough to identify statistically significant differences between the succeed and failed patients. For safety concern, we only used our biomimetic PLLA membrane in patients with non‐obliterated urethral stricture, which possess a well blood supply urethral bed. Thus, the indication of substitution urethroplasty using biomimetic PLLA membrane should be future investigated. Moreover, this finding requires confirmation in an adequately powered prospective randomized controlled trial with a long‐term follow‐up.

## 6 CONCLUSIONS

This study showed that the biomimetic PLLA membrane was a feasible and effective novel material for the anterior urethral repair. Urethral reconstruction using the biomimetic PLLA membrane should only be carefully considered with proper indications, including the stricture location, thickness of scar, and diameter of the remaining urethra lumen. Moreover, the long‐term effects with more patients should be investigated in further studies.

## CONFLICT OF INTEREST

Kunxue Deng, Jing Zhang, and Haitao Zhang are the employees of Medprin Regenerative Medical Technologies Co. Ltd. (Guangzhou, China), which is provider of the biomimetic poly‐l‐lactide (PLLA) nanofiber membrane. The other authors declare no competing interests.

## AUTHOR CONTRIBUTIONS

**Lujie Song:** Conceptualization (equal); data curation (equal); formal analysis (equal); methodology (equal); supervision (equal); validation (equal); writing – original draft (equal); writing – review and editing (equal). **Kunxue Deng:** Conceptualization (equal); formal analysis (equal); funding acquisition (equal); investigation (equal); resources (equal). **Wei Yuan:** Conceptualization (equal); data curation (equal); formal analysis (equal); writing – original draft (equal); writing – review and editing (equal). **Jing Zhang:** Conceptualization (equal); formal analysis (equal); investigation (equal); methodology (equal). **Jiahao Lin:** Conceptualization (equal); data curation (equal); formal analysis (equal); investigation (equal); visualization (equal). **Xiaoyong Hu:** Conceptualization (equal); data curation (equal); formal analysis (equal); methodology (equal). **Jianwen Huang:** Conceptualization (equal); data curation (equal); formal analysis (equal); methodology (equal). **Kaile Zhang:** Conceptualization (equal); data curation (equal); formal analysis (equal). **Haitao Zhang:** Conceptualization (equal); formal analysis (equal); investigation (equal); methodology (equal). **Jiemin Si:** Conceptualization (equal); data curation (equal); formal analysis (equal). **Hongbin Li:** Conceptualization (equal); data curation (equal); formal analysis (equal). **Tao Xu:** Conceptualization (equal); data curation (equal); formal analysis (equal); investigation (equal); methodology (equal); project administration (equal); supervision (equal); validation (equal); writing – original draft (equal); writing – review and editing (equal). **Qiang Fu:** Conceptualization (equal); data curation (equal); formal analysis (equal); funding acquisition (equal); methodology (equal); project administration (equal); resources (equal); supervision (equal); validation (equal); writing – original draft (equal); writing – review and editing (equal).

## Supporting information


**Appendix S1**. Supporting Information.

## Data Availability

The data that support the findings of this study are available from the corresponding author upon reasonable request.

## References

[1] Xu YM , Song LJ , Wang KJ , et al. Changing trends in the causes and management of male urethral stricture disease in China: an observational descriptive study from 13 centres. BJU Int. 2015;116:938‐944. doi:10.1111/bju.12945 25294184

[2] Lumen N , Hoebeke P , Willemsen P , De Troyer B , Pieters R , Oosterlinck W . Etiology of urethral stricture disease in the 21st century. J Urol. 2009;182:983‐987. doi:10.1016/j.juro.2009.05.023 19616805

[3] Barbagli G , Pellegrini G , Corradini F , et al. One‐stage penile urethroplasty using oral mucosal graft and glue. Eur Urol. 2016;70:1069‐1075. doi:10.1016/j.eururo.2016.04.025 27160949

[4] Xu YM , Qiao Y , Sa YL , et al. Substitution urethroplasty of complex and long‐segment urethral strictures: a rationale for procedure selection. Eur Urol. 2007;51:1093‐1098; discussion 8‐9. doi:10.1016/j.eururo.2006.11.039 17157433

[5] Song L , Murphy SV , Yang B , Xu Y , Zhang Y , Atala A . Bladder acellular matrix and its application in bladder augmentation. Tissue Eng Part B Rev. 2014;20:163‐172. doi:10.1089/ten.TEB.2013.0103 23895225

[6] Fu Q , Deng CL , Liu W , Cao YL . Urethral replacement using epidermal cell‐seeded tubular acellular bladder collagen matrix. BJU Int. 2007;99:1162‐1165. doi:10.1111/j.1464-410X.2006.06691.x 17244284

[7] Xu YM , Fu Q , Sa YL , Zhang J , Song LJ , Feng C . Outcome of small intestinal submucosa graft for repair of anterior urethral strictures. Int J Urol. 2013;20:622‐629. doi:10.1111/j.1442-2042.2012.03230.x 23131085

[8] Feng C , Xu YM , Fu Q , Zhu WD , Cui L . Reconstruction of three‐dimensional neourethra using lingual keratinocytes and corporal smooth muscle cells seeded acellular corporal spongiosum. Tissue Eng Part A. 2011;17:3011‐3019. doi:10.1089/ten.TEA.2011.0061 21736450

[9] Deng K , Yang Y , Ke Y , et al. A novel biomimetic composite substitute of PLLA/gelatin nanofiber membrane for dura repairing. Neurol Res. 2017;39:819‐829. doi:10.1080/01616412.2017.1348680 28701072

[10] Shi Z , Xu T , Yuan Y , et al. A new absorbable synthetic substitute with biomimetic design for Dural tissue repair. Artif Organs. 2016;40:403‐413. doi:10.1111/aor.12568 26526152

[11] Sun H , Lv H , Qiu F , et al. Clinical application of a 3D‐printed scaffold in chronic wound treatment: a case series. J Wound Care. 2018;27:262‐271. doi:10.12968/jowc.2018.27.5.262 29738294

[12] Li C , Xu YM , Song LJ , Fu Q , Cui L , Yin S . Urethral reconstruction using oral keratinocyte seeded bladder acellular matrix grafts. J Urol. 2008;180:1538‐1542. doi:10.1016/j.juro.2008.06.013 18710759

[13] Billiar K , Murray J , Laude D , Abraham G , Bachrach N . Effects of carbodiimide crosslinking conditions on the physical properties of laminated intestinal submucosa. J Biomed Mater Res. 2001;56:101‐108. doi:10.1002/1097-4636(200107)56:1<101::aid-jbm1074>3.0.co;2-6 11309796

[14] Lumen N , Vierstraete‐Verlinde S , Oosterlinck W , et al. Buccal versus lingual mucosa graft in anterior urethroplasty: a prospective comparison of surgical outcome and donor site morbidity. J Urol. 2016;195:112‐117. doi:10.1016/j.juro.2015.07.098 26241906

[15] Xu YM , Xu Q , Fu Q , et al. Oral complications after lingual mucosal graft harvesting for urethroplasty in 110 cases. BJU Int. 2011;108:140‐145. doi:10.1111/j.1464-410X.2010.09852.x 21091974

[16] Raya‐Rivera A , Esquiliano DR , Yoo JJ , Lopez‐Bayghen E , Soker S , Atala A . Tissue‐engineered autologous urethras for patients who need reconstruction: an observational study. Lancet. 2011;377:1175‐1182. doi:10.1016/s0140-6736(10)62354-9 21388673 PMC4005887

[17] Barbagli G , Akbarov I , Heidenreich A , et al. Anterior urethroplasty using a new tissue engineered oral mucosa graft: surgical techniques and outcomes. J Urol. 2018;200:448‐456. doi:10.1016/j.juro.2018.02.3102 29601924

[18] Ribeiro‐Filho LA , Sievert K‐D . Acellular matrix in urethral reconstruction. Adv Drug Del Rev. 2015;82‐83:38‐46. doi:10.1016/j.addr.2014.11.019 25477304

[19] Brown B , Lindberg K , Reing J , Stolz DB , Badylak SF . The basement membrane component of biologic scaffolds derived from extracellular matrix. Tissue Eng. 2006;12:519‐526. doi:10.1089/ten.2006.12.519 16579685

[20] Huang G , Li F , Zhao X , et al. Functional and biomimetic materials for engineering of the three‐dimensional cell microenvironment. Chem Rev. 2017;117:12764‐12850. doi:10.1021/acs.chemrev.7b00094 28991456 PMC6494624

[21] le Roux PJ . Endoscopic urethroplasty with unseeded small intestinal submucosa collagen matrix grafts: a pilot study. J Urol. 2005;173:140‐143. doi:10.1097/01.ju.0000146554.79487.7f 15592056

[22] Lumen N , Oosterlinck W , Hoebeke P . Urethral reconstruction using buccal mucosa or penile skin grafts: systematic review and meta‐analysis. Urol Int. 2012;89:387‐394. doi:10.1159/000341138 22889835

