# Supplementary material for: Anterior substitutional urethroplasty using a biomimetic poly‐l‐lactide nanofiber membrane: Preclinical and clinical outcomes
Source: Bioeng Transl Med. 2022 Mar 11;7(3):e10308. doi: 10.1002/btm2.10308 (PMC9472005; doi:10.1002/btm2.10308)
Supplement: Supplementary file 1 — Appendix S1. Supporting Information. [file BTM2-7-e10308-s001.docx]

**Supplementary Material**

**Anterior substitutional urethroplasty using a biomimetic poly-l-lactide nanofiber membrane: preclinical and clinical outcomes**

Lujie Song^1, #^, Kunxue Deng^2, #^, Wei Yuan^1, #^, Jing Zhang^2,3^, Jiahao Lin^1^, Xiaoyong Hu^1^, Jianwen Huang^1^, Kaile Zhang^1^, Haitao Zhang^2,3^, Jiemin Si^1^, Hongbin Li^1^, Tao Xu^*4,5^, Qiang Fu^*1^

1. Department of Urology, Shanghai Jiao Tong University Affiliated Sixth People's Hospital; Shanghai Oriental Institute for Urologic Reconstruction, Shanghai, 200233, China.

2. Medprin Regenerative Medical Technologies Co., Ltd., Guangzhou 510663, China

3. East China Institute of Digital Medical Engineering, Shangrao 334000, China

4. Bio-manufacturing Center, Department of Mechanical Engineering, Tsinghua University, 100084, China

5. Department of Precision Medicine and Healthcare, Tsinghua-Berkeley Shenzhen Institute, Shenzhen 518055, China

^#^Lujie Song, Kunxue Deng and Wei Yuan contributed equally.

^*^Corresponding author: Qiang Fu, MD., Department of Urology, Shanghai Jiao Tong University Affiliated Sixth People's Hospital, Shanghai, 200233, China. E-mail: jamesqfu@aliyun.com; Tao Xu, Bio-manufacturing Center, Department of Mechanical Engineering, Tsinghua University, Beijing, China. Email: drtaoxu@yeah.net


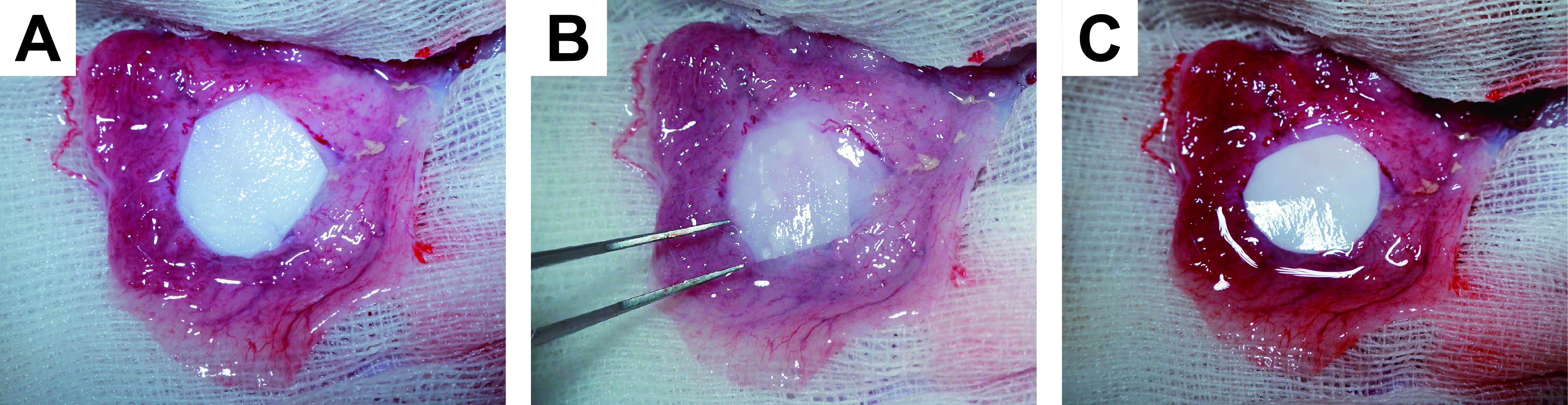


Figure S1. Compared with the bovine skin acellular matrix (A) and silk fibroin (B), PLLA nanofiber membrane (C) could attach smoothly on the surface of the rabbit bladder tissue.

1. Bovine skin acellular matrix is thicker and lack of flexibility.
2. Silk fibroin is thinner and friable.
3. PLLA nanofiber membrane is relatively flexible and have enough strength.


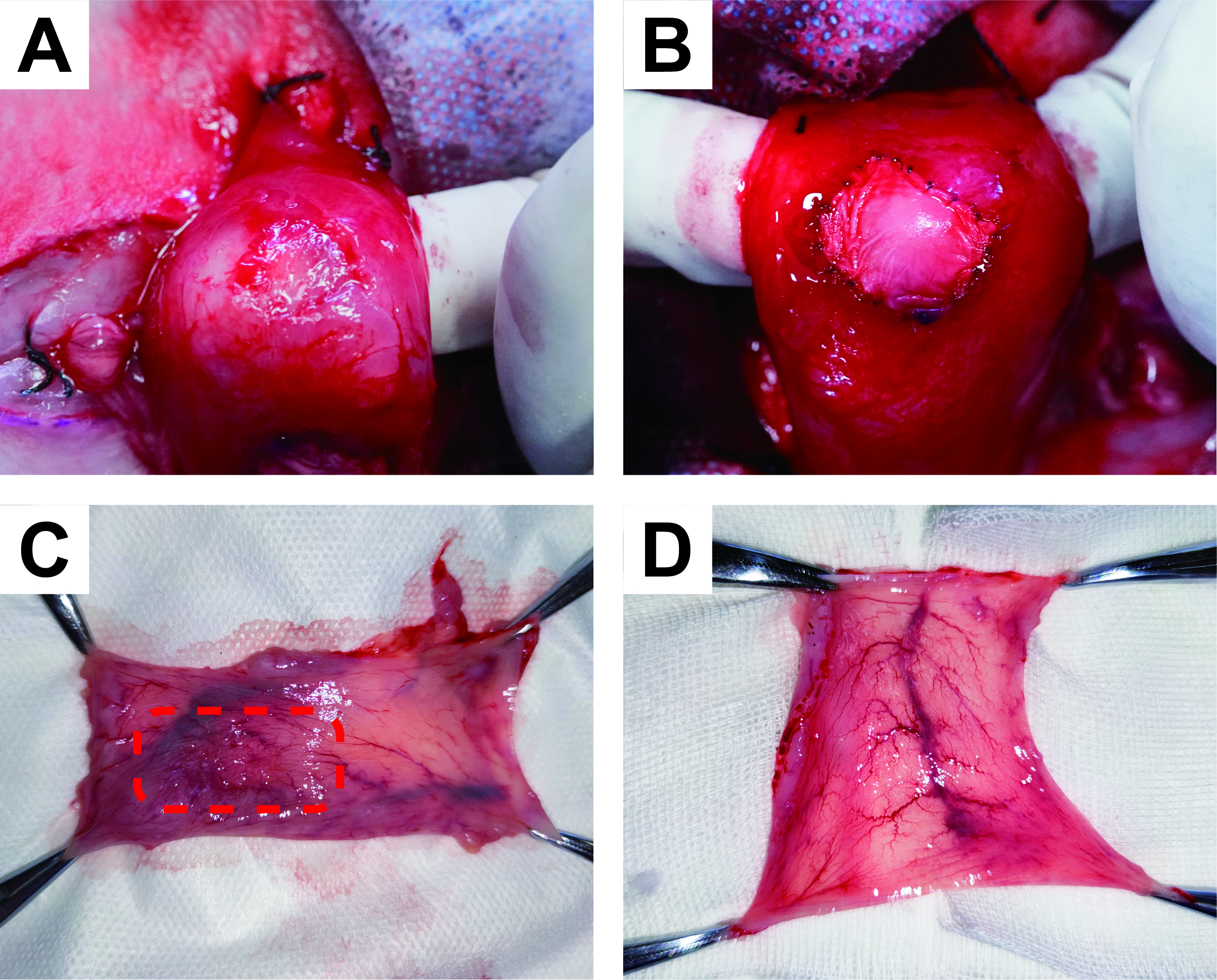


Figure S2. We created a bladder defect (A), then repaired with 1.5x1.0 cm biomimetic PLLA membrane (B). After two weeks, the animals were euthanized, from the represent gross observation, the fix area of bladder (C) was smooth and healthy as the normal bladder (D).


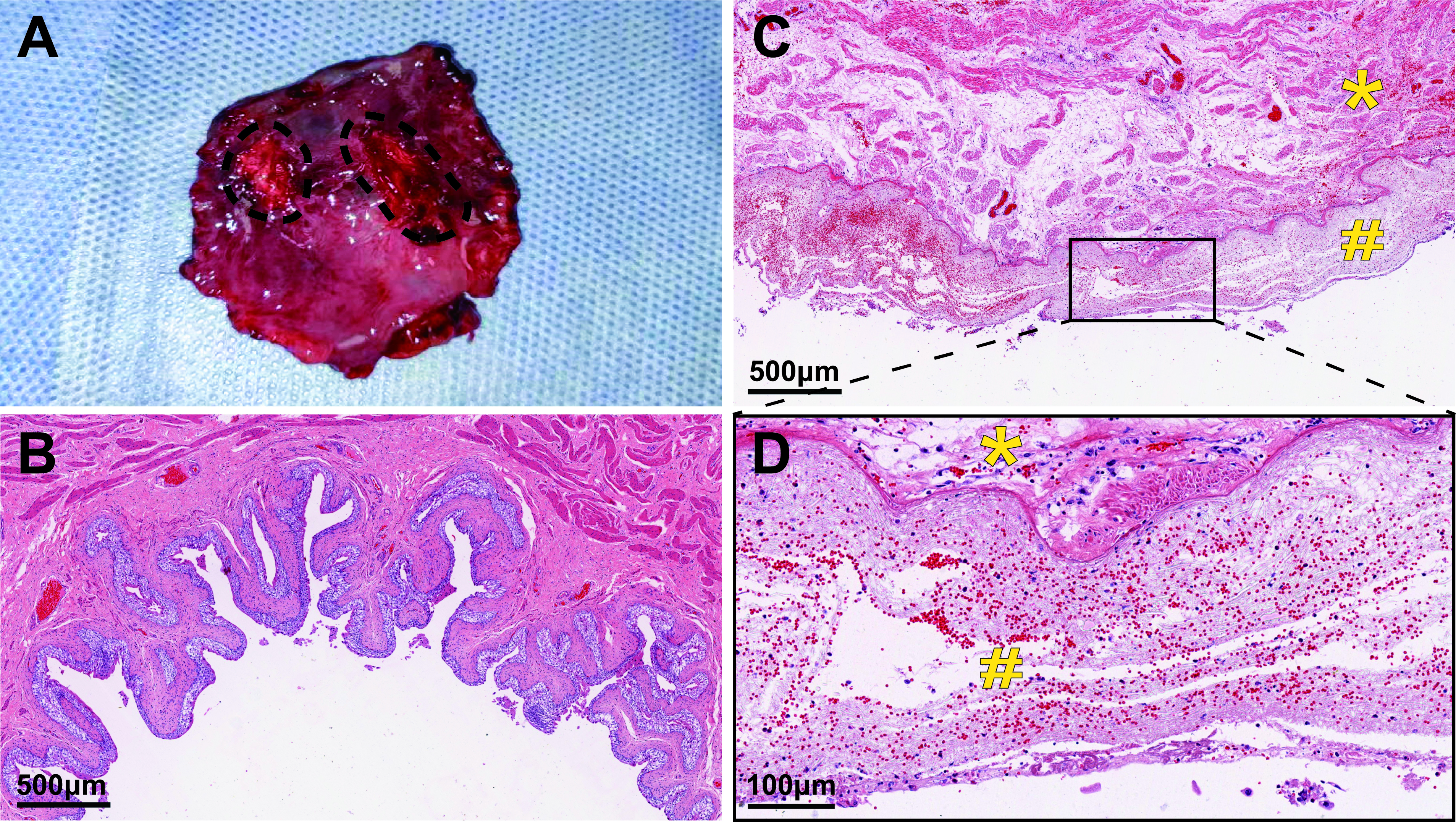


Figure S3. We used biomimetic PLLA membrane fixed the bladder defect. After 3 days, one of the animals was euthanized, from the represent gross observation (A), PLLA membrane attached smoothly and tightly on the surface of bladder (the fix area of bladder was marked with dotted line). We send both normal and fixed bladder for histological analysis. In hematoxylin and eosin staining (HE), compared with normal bladder tissue (B), the bladder defect lack of epithelium covering (C&D). The biomimetic PLLA membrane attached smoothly and tightly on the surface of the defect area. Lots of red blood cells and a few of white blood cells filled in the space of the biomimetic PLLA membrane (D). The yellow asterisk indicates the bladder tissue. The yellow number sign indicates the biomimetic PLLA membrane.


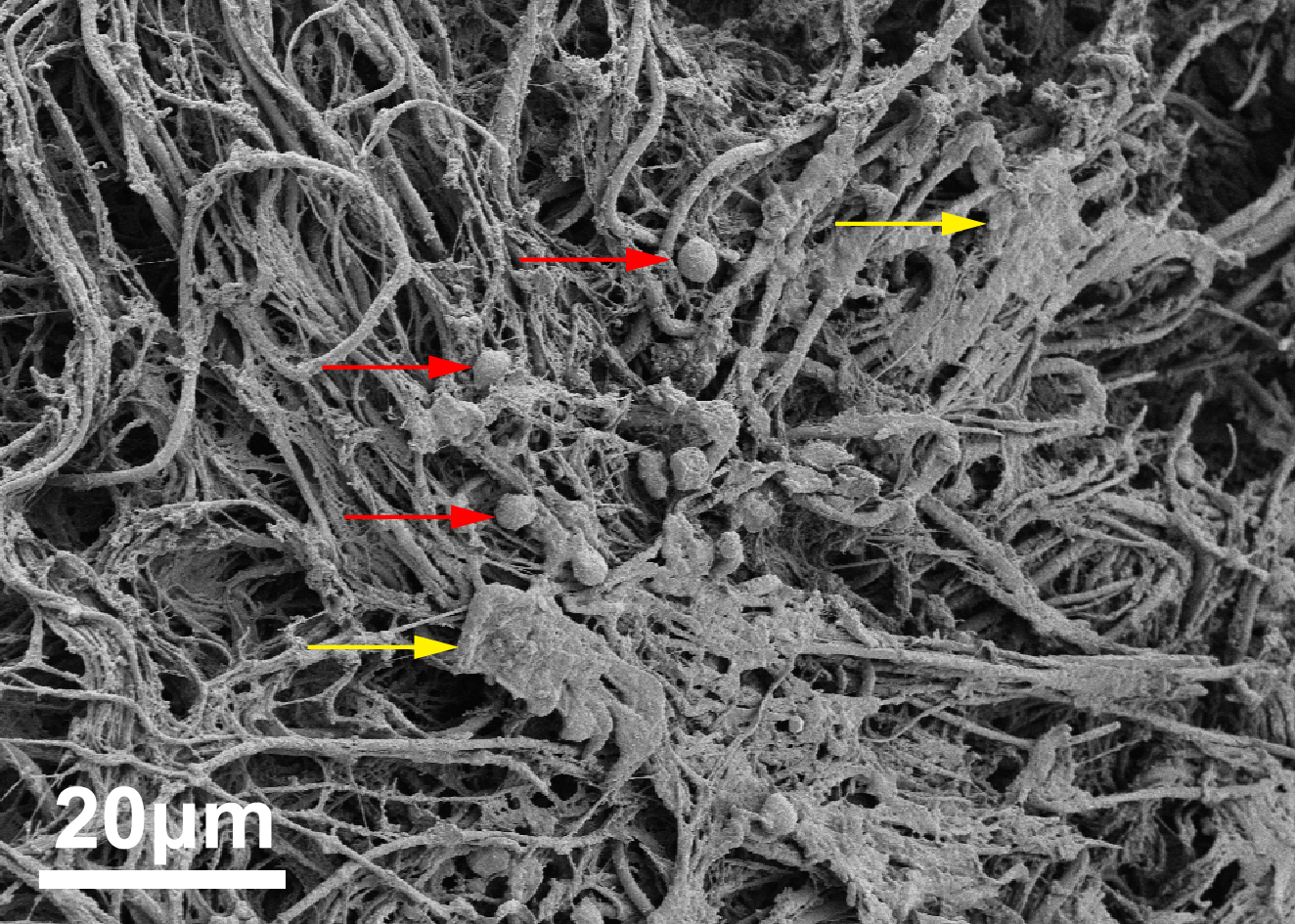


Figure S4. One of the tissues from the fixed area of bladder was analyzed by SEM. One of the represent images showed that the surface of the biomimetic PLLA membrane had been changed significantly compared with Figure 4A. Fibers of biomimetic PLLA membrane were bended and covered by secretions. Some cells could be found in the image, including red blood cells (pointed by red arrows) and other cells (pointed by yellow arrows) which could not be identified directly.





Figure S5. The stress–strain curves of the normal bladder (black line) and the postoperative bladder (red line).


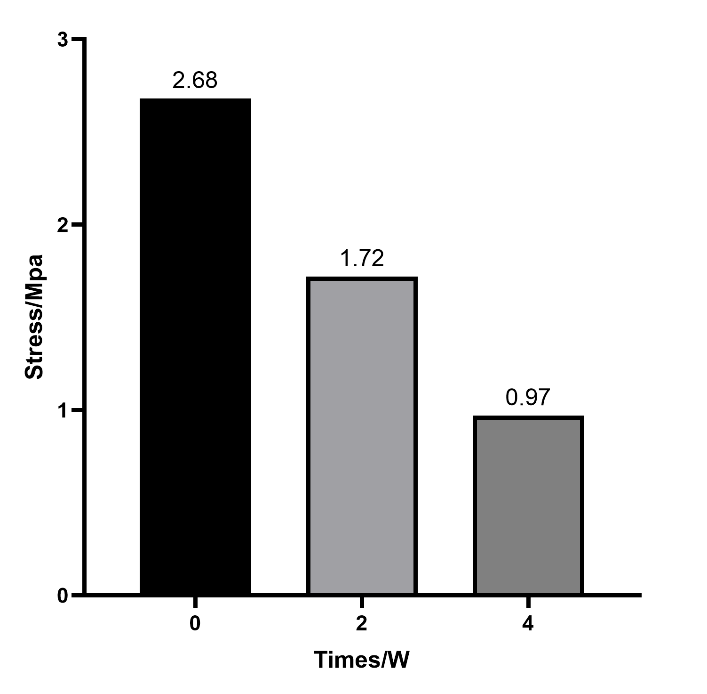


Figure S6. The in vitro degradation experiment of biomimetic PLLA membrane. The membrane was immerged in PBS solution, 50℃. The tensile strength test performed at 2 and 4weeks. The results showed that the initial tensile strength was 2.68 Mpa after 2 weeks and 1.72 Mpa after 2 weeks, and the loss rate was 35.76%. The tensile strength was 0.97 Mpa after 4 weeks, and the loss rate was 63.96%.
